# An Experimental and Clinical Research into Certain Problems Relating to Surgical Operations

**Published:** 1902-09

**Authors:** 


### BOOK REVIEWS.

An Experimental and Clinical Research into Certain Problems Relating to Surgical Operations. By Geo. W. Crile, A. M., M. D., Ph.D., Professor of Clinical Surgery, Medical Department Western Reserve University; Assistant Surgeon to Lakeside Hospital, Cleveland. Philadelphia: J. B. Lippincott & Co. 1901.

Dr. Crile has become so well known to the surgical and scientific world as a pioneer in physiological research, that the present monograph would naturally be expected to contain most interesting and suggestive material, and the fact that the work has received the Alvarenga prize for 1901 of the College of Physicians and Surgeons, amply attests this fact. Dr. Crile's original point of view and the methods by which he produces his work give exceptional value to all of his observations. Chapter III. on the effect of severing and mechanically irritating the vagi throws a most interesting light upon this much discussed question. It is of especial interest to surgeons to find that Dr. Crile, by actual experimentation, as well as by operations on living patients has determined that the results of this much feared accident in surgical operations is not nearly so serious as previously supposed, especially when the phenomena associated with the severance of these nerves are properly interpreted. Chapter IV. sheds an extremely interesting light upon the present views of intravenous infusion of saline solution. Chapter V. details a series of experiments with cocain solutions of varying strength. It is especially interesting to note that Dr. Crile's observations were largely made before the present general interest in this subject, and to observe that he has expressed himself with the greatest caution as to the ultimate use of spinal cocainisation. The present tendency toward a conservative estimate of the value of this procedure has been fairly foreshadowed by Dr. Crile's able exposition of the subject. Chapter VI. deals with the control of hemorrhage in operations about the head by temporary closure of the carotid arteries. Aside from the great value of this work as a stimulus to investigators, the facts gained by the experimental data are of the first importance and are modestly expressed by the author in the concluding remarks at the end of the book.

The proper interpretation of a slowed or of an accelerated pulse, or of an inhibited respiration, the prevention of either direct or reflex inhibition of the heart from mechanical stimulation of the vagus or its branches by the use of atropin and cocain, the safe and absolute control of hemorrhage by temporarily closing the carotid arteries render operative procedures of the head and neck so much safer as to greatly increase surgical possibilities."

